# Cylindroma of the breast with *CYLD* gene mutation: a case report and review of the literature

**DOI:** 10.1007/s11033-023-08606-y

**Published:** 2023-06-30

**Authors:** Vanessa Escher-Michlig, Tatjana Vlajnic, Luca Roma, Mattia Marinucci, Salvatore Piscuoglio, Matthias Matter, Martin Haug, Walter P. Weber, Simone Muenst

**Affiliations:** 1 Institute of Medical Genetics and Pathology, University Hospital Basel, Schoenbeinstrasse 40, 4031 Basel, Switzerland; 2 Visceral Surgery and Precision Medicine Research Laboratory, Department of Biomedicine, University of Basel, Basel, Switzerland; 3 Breast Center, University Hospital Basel, Basel, Switzerland

**Keywords:** Cylindroma of the breast, Cylindroma, Adenoid cystic carcinoma, Triple-negative breast tumor, Skin-adnexal type neoplasms

## Abstract

**Background:**

Cylindroma of the breast is a rare benign neoplasm. Since its first description in 2001, 20 cases have been reported in the literature.

**Methods and results:**

We report another case of this rare tumor in a 60-year-old woman with demonstration of the underlying molecular alteration. Histologically, the tumor showed the typical “jigsaw” pattern of a dual population of cells with a triple-negative phenotype. The pathognomonic mutation of the *CYLD* gene mutation was detected by whole exome sequencing. Cylindromas show morphological overlap with the solid-basaloid variant of adenoid cystic carcinoma, which renders this differential diagnosis difficult. However, distinction of these two lesions is of outmost importance, since cylindromas, in contrast to solid-basaloid variant of adenoid cystic carcinoma, behave in an entirely benign fashion.

**Conclusions:**

Careful evaluation of morphological features such as mitotic figures and cellular atypia is crucial in the diagnostic work-up of triple-negative breast lesions. It is important to keep cylindroma in mind as a pitfall and possible differential diagnosis for the solid-basaloid variant of adenoid cystic carcinoma. Molecular detection of *CYLD* gene mutation is helpful in cases with ambiguous histology. With this case report, we aim to contribute to a better understanding of mammary cylindroma and facilitate the diagnosis of this rare entity.

**Supplementary information:**

The online version contains supplementary material available at 10.1007/s11033-023-08606-y.

## Introduction

Cylindromas are rare and benign tumors of skin adnexal origin, commonly presenting as a single nodular lesion on the neck, head, or scalp [1]. The histogenesis of cylindromas is subject to debate including a possible follicular, apocrine, sebaceous, and eccrine origin [2]. Hair follicle stem cells are thought to give rise to cutaneous cylindroma [3]. Multiple cylindromas may arise in patients with *CYLD* cutaneous syndrome, including familial cylindromatosis, multiple familial trichoepithelioma and Brooke-Spiegler syndrome, which is characterized by a germline mutation within the tumor suppressor gene *CYLD* [3]. Several case reports describe cylindromas in unusual locations, including lung, kidney, salivary gland, and breast [4].

Cylindroma of the breast was first described by Gokaslan et al. in 2001 [5]. To the best of our knowledge, a total of twenty cases of cylindroma of the breast have been reported in the literature so far (Table 1) [4-16]. Only two of these cases occurred in patients with known *CYLD* cutaneous syndrome. [6, 7].

Here, we report another case of mammary cylindroma in a 60-year-old patient. For better understanding of this rare tumor entity, we conducted immunohistochemical (IHC), whole exome sequencing (WES), and fluorescence in situ hybridization (FISH) analysis. With this case report and the conducted review of the existing literature, we aim to highlight the importance of clinical findings, microscopic examination and molecular analysis to correctly distinguish between mammary cylindroma and solid-basaloid variant of adenoid-cystic carcinoma of the breast, its main differential diagnosis.

**Table 1:** Overview of reported mammary cylindromas in the literature [4-16]

Reference	n	Age	Diameter (mm)	Synchronous carcinoma	*CYLD* cutaneous syndrome	Clinical features	Molecular analysis	Therapy	Follow-up
[4, 5]	1	63	8	Infiltrating lobular carcinoma	No	Incidental finding in lumpectomy	Not performed	Local excision with axillary node dissection	Metastases of breast carcinoma No recurrence of cylindroma after 5 years
[4]	3	63–78	8–13	A infiltrating ductal carcinoma B, C no synchronous carcinoma	No	A incidental finding in lumpectomy B, C palpable masses	Not performed	A local excision with axillary node dissection B, C local excision	A metastases of breast carcinoma A-C no recurrence of cylindroma after 5 years.
[6]	4	37–85	7–13	A invasive ductal carcinoma and ductal carcinoma in situ B-D no	1	A Incidental finding in lumpectomy B-D screening mammography	Not performed	A bilateral mastectomy B-D local excision	A lost to follow-up B No recurrence after 2.5 years C No recurrence after 6 months D invasive ductal carcinoma after 18 years, no recurrence of cylindroma
[7]	1	59	5	No	1	Palpable mass	Not performed	Local excision	No recurrence after 2 years
[8]	1	62	16	No	No	Screening mammography	Not performed	Local excision	No recurrence after 6 months
[9]	1	41	15	No	No	Palpable mass	Not performed	Local excision	NA
[10]	1	61	20	No	No	Palpable mass	Not performed	Local excision	No recurrence after 12 months
[11]	1	72	20	No	No	Palpable mass	Not performed	Wide local excision with axillary node biopsy	No recurrence after 7 months
[12]^c^	3	NA	7–12	A Grade 2 carcinoma NST^b^ B, C no synchronous carcinoma	No	A Incidental finding in mastectomy B, C screening mammography	Not performed	A mastectomy B, C local excision	A NA B No recurrence after 15 months C No recurrence after 22 months
[13]	1	66	9	No	No	Screening mammography	Somatic *CYLD* splice site mutation (c.1890 + 2T > C) absence of MYB-NFIB fusion	Quadrantectomy with sentinel lymph node biopsy	No recurrence after 37 months
[14]	1	55	8	No	No	Screening mammography	Not performed for *CYLD* mutation. *PMS2* mutation linked to Lynch syndrome	Local excision with adjuvant radiation therapy	No recurrence after 4 months
[15]	1	71	25	No	No	Palpable mass	Not performed	Local excision	NA
[16]	1	80	50	No	No	Palpable mass	Not performed	Radical mastectomy	No recurrence after 27 months
Current case	1	60	8	No	No	Screening mammography-	Missense mutation of *CYLD*: c.2273G > A (p.Arg758Gln) absence of *MYB-NFIB* fusion	Local excision	No recurrence after 24 months

^a^ *NA* not available; ^b^ *NST* of no special type; ^c^ Cases A and C were also described as “low-grade salivary gland like carcinoma with predominant cylindromatous differentiation” [12]

## Materials and methods

### Immunohistochemistry

The resected tissue specimen was formalin-fixed and paraffin-embedded (FFPE) according to standard procedures. IHC was performed on 4 μm thick sections, using the Ventana Benchmark XT Ultra automated staining system (Ventana Medical Systems, Tucson, Arizona). Primary antibodies listed in Table 2 were detected using the OptiView DAB IHC Detection Kit (Ventana Medical Systems) by incubation with OptiView HQ Universal Linker, OptiView HRO Multimer, OptiView Amplifier, and OptiView Amplifier Multimer for 12 min each. Slides were counterstained with hematoxylin and Bluing reagent for 4 min each.

**Table 2:** Immunohistochemical antibodies

Protein	Code number	Clone	Dilution	Pretreatment (min)	AB inc. time (min)
ER	Ventana 790–4324	SP1	RTU^a^	CC1 24	8
PR	Ventana 790–4296	1E2	RTU	CC1 24	4
Her2	Ventana 790–2991	4B5	RTU	CC1 32	16
CK7	Ventana 790–4462	SP52	RTU	CC1 32	16
P63	Ventana 790–4509	4A4	RTU	CC1 32	32
CD117	Cellmarque 117R	Poly	1:50	CC1 40	16
Ki67	Dako IR626	Mib1	RTU	CC1 32	16

^a^*RTU* Ready to use

### Fluorescence in situ hybridization

For fluorescence in situ hybridization, a SPEC MYB Dual Color Break Apart Probe (Zytovision, Bremerhaven, Germany; Cat-No. Z-2143-200,) was used for the detection of specific translocations involving the human *MYB* gene at 6q23.3. The orange fluorochrome direct labeled probe hybridizes distal, the green fluorochrome direct labeled probe hybridizes proximal to the breakpoint region of the *MYB* gene. For visualization of the probes, a 4 μm thick FFPE slide was deparaffinized, pretreated, and hybridized overnight.

### DNA extraction and whole exome sequencing

DNA from fresh-frozen tumor and formalin-fixed paraffin-embedded normal tissues was extracted using DNeasy blood and tissue kit (Qiagen, Germantown, MD, USA) according to the manufacturer’s instructions and quantified using the Qubit Fluorometer assay (Life Technologies, Carlsbad, CA, USA), according to manufacturer’s guidelines. Twist Human Core Exome Plus kit (Twist Bioscience) panel was utilized for the whole exome capturing.

Paired-end 100-bp reads were generated on the Illumina NovaSeq 6000. Sequencing was conducted by CeGaT (Tübingen, Germany).

Reads obtained were aligned to the reference human genome GRCh38 using Burrows-Wheeler Aligner (BWA, v0.7.12) [17]. Local realignment, duplicate removal, and base quality adjustment were performed using the Genome Analysis Toolkit (GATK, v4.1 and Picard)^1^ .

Somatic single nucleotide variants (SNVs) and small insertion and deletions were called using MuTect2 (GATK 4.1.4.1) [18]. All SNVs with variant allelic fractions less than 1% or that were supported by fewer than 3 reads were discarded to reduce false positive results. We further excluded variants identified in at least two of a panel of 123 non-tumor samples, including the non-tumor samples included in our study. Variant annotation was performed using SnpEff software v4.1 [19]. FACETS v0.5.14 enabled the identification of allele-specific CNAs [20].

### Case presentation

The 60-year-old patient presented with a small nodule of about 5 mm size in her left breast detected on mammography. There were neither skin nor nipple changes, and there was no sign of an axillary lymphadenopathy. Her personal history included an invasive carcinoma of no special type (NST) with lymph node metastasis 9 years earlier in the contralateral breast, which had been treated with neoadjuvant chemotherapy followed by mastectomy and axillary dissection as well as adjuvant radiotherapy. Two years later, she had undergone reconstructive surgery on the right breast. Otherwise, the personal history was unremarkable, with several benign naevi, seborrheic keratosis, and a histiocytoma of the skin.

### Core needle biopsy specimen

**Fig. 1:**
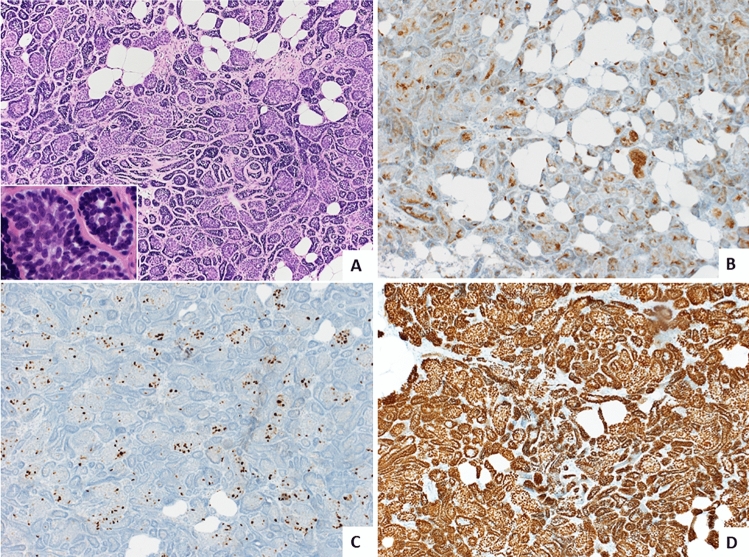
**A **Biopsy specimen showing the typical ”jigsaw” pattern of cell nests, containing a dual population of larger, eosinophilic, epithelial cells in the center and smaller, basaloid, myoepithelial cells in the periphery (H&E, magnification 100x). **B **Immunohistochemical analysis for CD117/c-kit (magnification 100×) showing positivity of the inner epithelial cells for CD117, while the outer myoepithelial cells remain negative. **C **Immunohistochemical analysis for Ki-67 (magnification 100x) showing a very low proliferation fraction of about 5–10%. **D **Immunohistochemical analysis for p63 (magnification 100×) showing diffuse and strong positivity for this myoepithelial cell marker in all tumor cells. (Color figure online)

**Fig. 2:**
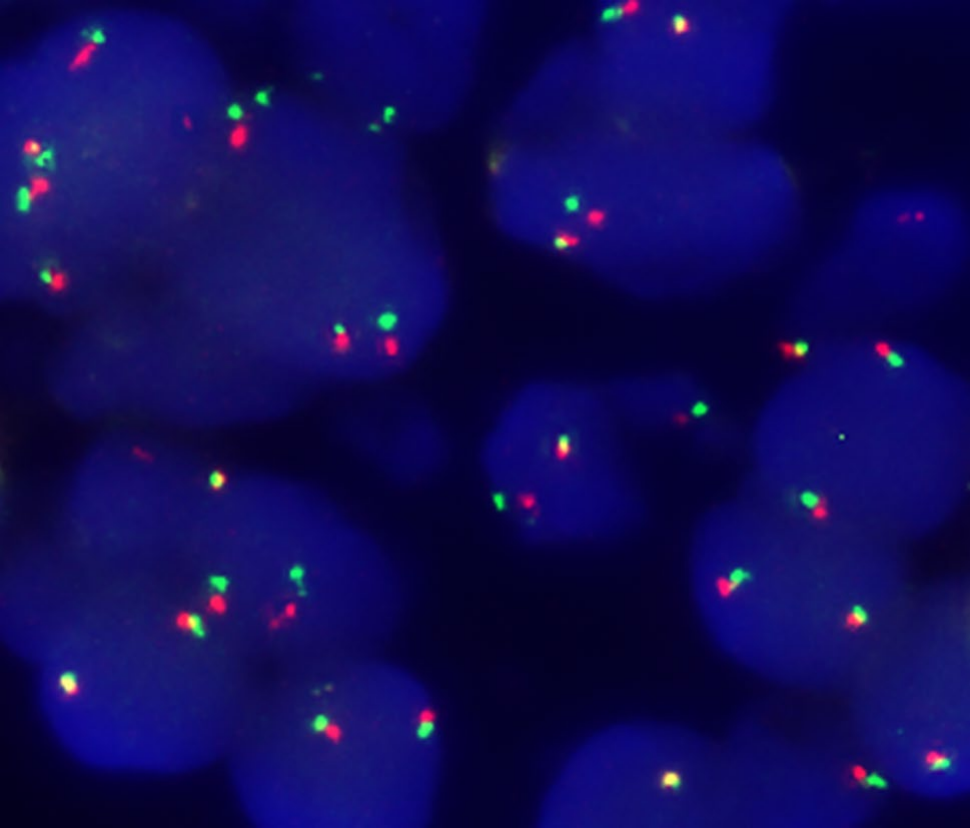
*MYB* fluorescence in situ hybridization (FISH) showing adjacent or fused signals of the 3′ and 5′ probes, indicating absence of the *MYB-NFIB* fusion gene (green and orange signals, respectively, original magnification 600x). (Color figure online)

**Fig. 3:**
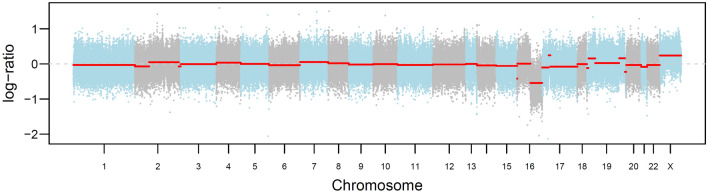
Loss of heterozygosity (LOH) of 16q with the *CYLD* locus wild type allele. (Color figure online)

To rule out a second malignancy, sonographically guided core needle biopsy (CNB) of the nodule was performed. Histologically, a non-encapsulated tumor composed of cell nests arranged in a distinct “jigsaw” pattern was visible on all four cores (Fig. 1A). The lesion seemed to have an irregular border, with cell nests infiltrating the adjacent adipose tissue, mimicking an infiltrative growth. The cell nests consisted of a dual population of cells, including central epithelial cells with increased eosinophilic cytoplasm and peripheral myoepithelial basaloid cells with hyperchromatic oval nuclei arranged in a palisading pattern. Each nest of cells was surrounded by a strongly periodic-acid Schiff (PAS) positive basement membrane. Importantly, no cellular atypia or increased mitoses were identified. Immunohistochemically, the tumor showed expression of estrogen and progesterone receptors in very few (< 1%) cells while being negative (1+) for Her2/neu. The proliferation index (Ki-67) was around 5–10% (Fig. 1C). C-kit (CD117) as well as CK7 were positive in the central epithelial cells (Fig. 1B), while the myoepithelial marker p63 showed diffuse and strong positivity in all tumor cells (Fig. 1D). To exclude the differential diagnosis of solid-basaloid variant of AdCC, a FISH for detection of the *MYB-NFIB* fusion gene t(6; 9) (q22:p23) was performed and showed no break apart of the *MYB* gene (Fig. 2). However, a distinct mutation of the tumor suppressor gene *CYLD* (missense mutation c.2273G > A, p.Arg758Gln) was found by WES (Suppl. 1). In addition, there was loss of heterozygosity (LOH) of 16q, with the *CYLD* locus wild type allele (Fig. 3). In view of all these results, the diagnosis of cylindroma of the breast was made, and the breast biopsy was classified as B3.

### Excisional biopsy specimen

The patient subsequently underwent excision of the lesion without sentinel lymph node biopsy. In the excision specimen, the cylindroma showed a total size of 8 mm and the same morphology as in the CNB, with irregular margins and cell nests pushing into the adipose tissue, thereby mimicking an infiltrative growth. The tumor was clearly located within breast tissue, not attached to the skin.

24 months after surgery, the patient remains free of disease.

## Results and discussion

Adnexal tumors arising within the breast are very rare. In our literature search, we found 20 reported cases of cylindroma of the breast, all described in female patients (Table 1). Two of these cases occurred in the context of familial cylindromatosis, one in context of Lynch syndrome and the remaining 17 patients presented with isolated breast lesions. So far, no recurrences or distant metastases have been reported in the literature, confirming the notion that cylindroma of the breast is a benign lesion. Thus, there seems to be no necessity for wide local excision or axillary lymph node excision. However, a correct diagnosis of the lesion is essential for the choice of adequate therapy.

Histologically, cylindroma of the breast typically consists of inner epithelial and outer myoepithelial cells arranged in tightly packed cell islands to form the typical “jigsaw” or “mosaic” pattern. Each island is surrounded by a dense band of hyaline tissue, which is strongly PAS positive. Molecular analysis typically shows a distinct mutation of the *CYLD* gene [13] (in our case missense mutation p.Arg758Gln).

The main histological differential diagnosis of cylindroma of the breast is the solid-basaloid variant of AdCC. This entity shows a more aggressive behavior with local recurrences and the possibility of distant metastases, requiring a more intensive treatment [21]. Differentiation between these two lesions can be challenging, since they are both triple-negative, and consist of a dual population of cells, including CK7 and C-kit (CD117) positive central epithelial and p63 positive peripheral myoepithelial cells. Important morphological differences between mammary cylindroma and solid-basaloid variant of AdCC of the breast include marked cellular atypia and mitotic figures in the latter. Moreover, almost all cases of AdCC show the distinct *MYB-NFIB* gene fusion, which has never been shown in mammary cylindroma, to the best of our knowledge. Peculiarly, recurrent *MYB-NFIB* gene fusion has been reported in a subset of dermal cylindromas [22]. From the data presented, we cannot exclude the possibility that some of these cylindromas may actually represent AdCC. As histological and immunohistochemical features can be overlapping, distinction between these differential diagnoses requires special attention to subtle differences such as mitotic figures and cytological atypia, which was not specifically addressed in this study.

The low incidence of mammary cylindroma is possibly because in the past some cylindromas may have been misclassified as other basaloid tumors, such as AdCC, syringoma or other salivary gland type tumors due to overlapping morphological and immunohistochemical features. An interesting theory by Senger et al. suggests that a common pluripotent progressor cell might give rise to breast neoplasms with the ability to differentiate into epithelial and myoepithelial cells, such as breast adenomyoepithelioma and breast cylindroma or malignant lesions, including low-grade adeno-squamous carcinoma, squamous carcinoma, metaplastic carcinoma, AdCC and the solid-basaloid variant of AdCC [23].

On the other hand, a distinct somatic or germline mutation in *CYLD* is typical for cylindroma. *CYLD* is a tumor suppressor gene located on the 16q12.1 locus [13]. It plays a role in the post-translational modification of proteins, which is important for DNA repair, endocytosis, proteosomal degradation and the regulation of several cell-signalling pathways, such as NF-kB, Wnt, Notch, and TGFB1 signaling pathway [3]. To date, over 100 mutations of this tumor suppressor gene have been reported, most frequently occurring in cylindromas [3]. Somatic *CYLD* mutation and LOH of the *CYLD* locus wild type allele was associated with a sporadic mammary cylindroma [13], while a germline mutation typically results in familial cylindromatosis, multiple familial trichoepitheliomas and Brooke-Spiegler syndrome [3]. So far, only one specific *CYLD* mutation, a somatic *CYLD* splice site mutation (c.1890 + 2T > C), has been reported by Fusco et al. in breast cylindroma [13]. In our case, we found a missense mutation of *CYLD* p.Arg758Gln, which represents a new *CYLD* mutation in this tumor. Importantly, a malignant transformation to cylindrocarcinoma has only been described for dermal cylindroma in the context of *CYLD* cutaneous syndrome, but there have been no reports of malignant transformation in breast cylindroma [3].

## Conclusion

In summary, our case shows the typical histological and immunohistochemical features of a rare mammary cylindroma, which was confirmed by molecular analysis. When dealing with triple-negative breast tumors in daily diagnostic practice, it is important to keep cylindroma in mind as a pitfall and a possible differential diagnosis for the solid-basaloid variant of AdCC in order to avoid overtreatment. Careful evaluation of morphological features such as mitotic figures and cellular atypia is crucial in the diagnostic work-up of triple-negative breast lesions, and molecular detection of *CYLD* gene mutation is an important diagnostic adjunct in cases with ambiguous histology.

## Supplementary information

Below is the link to the electronic supplementary material.

Supplementary material 1 (XLSX 12.7 kb)

## Data Availability

All the data are available on request.

## Abbreviations

AdCC: Adenoid cyctic carcinoma
IHC: Immunohistochemistry
WES: Whole exome sequencing
FISH: Fluorescence in situ hybridization
SNVs: Somatic single nucleotide variants
VAFs: Variant allelic fractions
NST: No special type
CNB: Core needle biopsy
PAS: Periodic-acid Schiff
LOH: Loss of heterozygosity

## Footnotes

1 http://broadinstitute.github.io/picard/.
